# The New York Institute of Stomatology

**Published:** 1899-12

**Authors:** 

**Affiliations:** The New York Institute of Stomatology


### THE NEW YORK INSTITUTE OF STOMATOLOGY.

A REGULAR meeting of the Institute was held on Tuesday evening, June 13, 1899, at the office of Dr. Chas. D. Cook, No. 162 Remsen Street, Brooklyn, the President, Dr. E. A. Bogue, in the chair.

The minutes of the last meeting were read and approved.

### SPECIAL TOPIC: EROSION.

Dr. Wm. H. Potter, of Boston, Mass., read a *résumé* of a paper by Dr. M. Michaels, entitled "Du rôle de l'hyperacidité organique et des sulfocyanures salivaires dans l'abraison chimique des dents."

*Dr. Potter.*—In preparing a *résumé* of the paper under consideration two courses suggested themselves to me. One was to present the contents of the paper briefly in my own language, and the other to present literal translations of the most important parts. I chose the latter course, thinking that by so doing I could more accurately present the writer's thoughts, though I realized that a *résumé* prepared in this way was in danger of seeming fragmentary and without the accurate sequence which would be found in a paraphrase.

The author begins with a description of the "acid diathesis," so called, going into the physiological chemistry of the subject. He quotes from M. Gautrelet to the effect that "alkalinity promotes organic oxidations and acidity diminishes them." There follow charts showing the increased acidity as shown in the urinary analyses of gouty and rheumatic subjects. As to the dental manifestations of this diathesis let me quote literally, as follows:

"By the side of the collection of types which characterize this acid diathesis we sometimes observe characteristic accidental symptoms affecting the dental appliances of the buccal mucous membrane, as, for instance, arthritic gingivitis, alveolar desmodynia, pain in a ligament, buccal acidity, loosening of the teeth, nervous constriction of the palate, alveolar-dental pyorrhea, expulsive gingivitis, chemical abrasion, deposit of sordes, dental tartar, and the caries diathesis.

"As a result of my observations on affections of the buccal mucous membrane, and on certain characteristics of dental caries shown in the mouths of the hyperacids, I have been led to study these characteristics varying from individual to individual.

"I have been able in the same course of investigation to study the chemical composition of the saliva and also of other secretions, as the sweat and urine. In the urinary and salivary analyses of subjects presenting chemical abrasion of the teeth the rate of total acidity was less than with the out-and-out hyperacids, like those having gout and rheumatism: uric acid was even wanting in the polariscope examination; on the contrary, oxalic acid was present in these urines in the form of crystallized oxalate of lime. Sulphocyanide of potassium or ammonium was present in the salivary secretions in a quantity more than normal. With these individuals the characteristic manifestations about the gums were absent, but abrasion of the teeth was manifest. I do not wish to decide on the spot whether the salivary glands secrete an increased amount of sulphocyanides, as these principles exist always in the saliva of people who do not offer the characteristic alteration of chemical abrasions."

Here we have, then, the contents of the essay pretty well defined. The existence of the acid diathesis is a well-proved fact, and its connection in a causative way with gout and rheumatism seems very well assured. Dental lesions are also noticed where this diathesis exists, and also the presence of an increased amount of urates and sulphocyanides of ammonium and potassium in the saliva.

The object of the writer is to connect certain dental lesions, and in particular so-called chemical erosion with the presence of an increased amount of sulphocyanides in the saliva, an increase which he claims is always noted in cases of acid diathesis.

In the next section the author quotes from Dr. Etchepareborda, of Buenos Ayres, as to "the influence of rheumatism on the production of diseases of the mouth and dental system." Dr. Etchepareborda has gathered together ninety observations of rheumatic cases, in which he finds the following buccal or dental accidents: Twenty-five cases of simple alveolar absorption; forty cases of simple alveolar absorption with gingivitis; fifteen cases of simple alveolar absorption with gingivitis and osteo-periostitis; eight cases of spontaneous loss of teeth; two cases of caries with alveolar-dental periostitis. The conclusions of the observer quoted were as follows:

I. The teeth, the maxillaries, and the soft parts of the mouth are often the seat of accidents of rheumatic origin.

II. These accidents may precede, accompany, or follow articular manifestations. They can long remain isolated and constitute the only visible expression of the diathesis.

III. The most frequent accidents of rheumatic origin are the following:

1. As regards the teeth: alveolar periostitis, alveolar-dental periostitis, dental necrosis, and the spontaneous falling of the teeth.

2. As regards the gums: simple or aphthous inflammations.

3. As regards the nervous system: facial neuralgia.

4. As regards the maxillary: alveolar resorption, caries, and necrosis of the maxillary bones.

IV. No one of these local affections can positively be regarded as characteristic of rheumatism. It is impossible to fix their place in the chronology, and the order of succession of rheumatic accidents.

There then follows a description from Bödecker of the characteristics of erosion. The author then adds:

"I desire to complete this description by drawing attention to certain characteristics of this lesion, particularly in relation with the labial glands. The characteristics of chemical abrasions vary infinitely both in form and depth. But their situation always depends on that of the labial glands, which, as we will see, are the organs secreting, in the pathological conditions which we will determine, the active principle which produces abrasion. It is possible to describe a type of this lesion, which according to my observations is the following:

"The intermittent contact of the glandular secretion with the surface of the tooth gradually dissolves the enamel. The lesion does not spread at first, but scoops out the tissue, since it depends essentially on the situation of the secreting gland.

"At this stage, and according to types which I possess, the abraded surface presents jagged edges, the bottom is smooth, and the tissues have preserved their color. In course of time the hollowed cavity increases in depth and sometimes mines the tissues under the enamel. In these cases the active principle spreads in this direction and so produces the lesion. Usually the sides of the abrasion are sharply cut, although in some cases they are more or less dull.

"No form of caries presents a like appearance, and the two processes cannot be confused. The affected teeth are more or less in number. Sometimes one or two may present the abrasion, in other cases a large number. The duration of the destructive process is relatively long. The active principle is secreted in infinitesimal quantities, and, moreover, the contact between the tooth and the orifice of the labial glands is intermittent.

"After what has been said above, of diathesis in general and of the changes which they impose upon organic centres, it is in the saliva that we must seek for the cause of this lesion.

"The situation of the duct of the labial glands must be relied upon to explain the localization of abrasion. The labial mucous glands are situated between the muscular layer of the orbicularis of the lips and the mucous membrane which covers them. They are very numerous, and form a complete ring about the buccal orifice. They are grape-shaped glands, furnished with a main excretory canal enlarged at its inferior extremity and opening at the surface of the mucous membrane into the vestibular cavity; often the principle excretory duct receives the excretory conduits of small mucous glands.

"In order to examine these glandular orifices, you raise the lips, invert them, and dry the surface. At the end of a minute you see a tiny drop of the liquid which they secrete. The saliva produced by a mixed secretion of the salivary glands is slightly alkaline, and contains a distinctive ferment,—ptyaline,—in the proportion of 7 to 1000. Besides, it contains mucin, chloride of sodium and potassium in variable quantities, sulphates of sodium and potassium lactates, earthy phosphates and carbonates, phosphate of iron, fatty matters, and uric acid derivatives. These are constant elements. But we can also find there in certain conditions, urea, glucose, biliary pigments, lactic acid, and leucine. The fatty acids, acid urates, lactic acid, oxalic acid, and the sulphocyanides represent the acid factors having a great affinity for the chalky base of the tooth. Chemical analyses of the saliva made by the reaction of perchloride of iron show the presence of the sulphocyanides. I have sought to produce experimentally the lesions of abrasion. The theoretical action of the alkaline sulphocyanides (of potassium and ammonium) is the following: They dissolve the organic elements of the dental organs and lay bare the mineral elements, forming with them a sulphocyanide of calcium and soluble phosphates of potassium and ammonium."

Here is a practical test which the author has made. A patient whose anterior teeth showed extensive and characteristic erosions was examined by a chemist with regard to saliva and urine. This examination showed a saliva of neutral reaction possessing double the amount of sulphocyanides to be found in normal saliva. The sulphocyanides were ammoniacal salts, and not potassium salts as found in normal saliva. The urinary analysis made at the same time gave the signs which characterize the rheumatic diathesis.

In another case, where there was extensive abrasion and extreme sensitiveness of the abraded surfaces, the tests for sulphocyanides showed a small amount in the saliva, but a strong ammoniacal reaction. This case was treated by chemical cautery, with great relief to the patient. The author then enumerates the various cauterants which have been used in the treatments of erosions, and recommends the chloride of antimony ($SbCl_3$). He directs its use as follows: "Turn back the lip and protect the mucous membrane with a pad of cotton. Dry the surface of the teeth and take in the blunt end of a quill toothpick a small drop of chloride of antimony. Bathe the surface of the erosion, taking care not to touch the gums, and immediately place over the tooth a bit of German tinder of suitable size, which is to be kept in position for some seconds. Finally, wash the mouth with bicarbonate solution. The chemical action of the antimony chloride is often quite painful, but the pain is always of short duration. As to the prophylaxis of chemical abrasion, it consists in a local treatment and in a general one calculated to modify the diathesis. It is possible to stop tooth-destruction by ignipuncture of the labial glands, using a thermo-cautery. These glands are very small and superficial; the least cauterization will destroy them. Moreover, persons suffering from hyperacidity, by modifying their *régime* under an alkaline treatment and by promoting oxidization, will modify their physiological chemistry."

In the closing section the author describes his experiments with which to imitate out of the mouth the chemical process which he claims to be going on in the mouth. A tooth is subjected to a slow drip of a solution of sulphocyanide of potassium, and in a few days' time the enamel presents abrasions exactly like those seen in the mouths of individuals having chemical erosion.

*The President.*—Dr. Dawbarn, who is to present the case upon which he operated for the removal of the superior maxilla, has arrived, and, in order that he may not keep his patient waiting unnecessarily, this portion of the programme will be taken up before the discussion of Dr. Potter's paper.

*Dr. Robert H. M. Dawbarn.*—The patient which I am to present to you to-night was shown to the Institute of Stomatology at its April meeting. The case is one originally of sarcoma of the antrum. At that time quite a number of the members who are here to-night examined her, and several honored me by being present at the subsequent operation.

(At the time when the patient was first presented to you there was a large tumor filling the antrum and causing a decalcification of the superior maxilla. The interesting test of passing a needle through this softened bone was performed at my request by your President.)

She was first taken by her physician to the surgical section of the New York Academy of Medicine, where this diagnosis was made by the surgeons present; and knowing that I am especially interested in this line of work, Dr. Freudenthal sent her to me.

The operation which was performed in this case is called excision of the superior maxilla, but in reality it is a removal not only of the whole superior maxilla, but also of part of the nasal and lachrymal, the entire inferior turbinated, and about one-half of the malar and palate bones.

The incision adopted runs up the middle of the lip, thence along the naso-facial junction, and then follows a line somewhat below the lower border of the orbit, out onto the prominence of the cheek. Within the mouth it runs from between the central incisors, directly backward to the junction of the hard with the soft palate; then transversely outward to the gum, behind the last molar, being careful not to sacrifice the soft palate with the hard. The soft parts are reflected, and the bones are then sawed and chiselled until free. In this case the bone was so softened from disease that it came away in numerous fragments, and not in one piece.

It is doubtful if I would have been able to perform this operation at all, because of the unusual vascularity, had I not as a preliminary step ligated both external carotids. In this way it was possible to control in great measure the hemorrhage; and even then it was necessary before the end of the operation to use intravenous saline infusion to the extent of two litres. The results, so far as the operation itself was concerned, were excellent. Of course it is to be expected that there will be some sinking in of the soft parts, but by no means so great as would be supposed from the enormous mass of bone which has been removed. A good prosthetical appliance in the shape of a dental half-set, with a plumper for the cheek, will go far to restore both voice and normal appearance.

About a week after the operation I noticed, as a new development upon the operated side, a little swelling of the eyelids. Upon examining I found by very deep pressure a lump, which seemed quite firm, growing from the back of the orbit. It was evident to me that this growth must be of the same nature,—a round-cell sarcoma. The patient willingly submitted to another operation, and this was performed without delay. The right eyebrow was shaved, and the incision ran the length of it and then down the nose half-way. Upon turning back the flap a growth was found on the inner wall of the orbit about the size of a chestnut, and attached so far back that after its removal the optic nerve was easily seen. This tumor being removed, I was about to close the wound when it occurred to me that, it being very near the frontal sinus, the latter also might be involved. I therefore drilled into it and found my surmise was correct: it was solidly filled with the growth. To meet this rare and dreadful complication I had to drill away the entire front wall of the frontal sinus and thoroughly use the sharp curette. As a natural consequence of this operation it would be supposed that the eyeball would turn outward, the superior and inferior oblique and internal rectus muscles having been involved and cut. But a week or two afterwards I found that the eyeball was turning inward. This led me to suspect further involvement within the outer side of the orbit, and upon examination by deep palpation this was found to be the case. Since its discovery it has grown very rapidly. It is a pitiable condition, and the patient's only hope is in another operation whereby this third tumor shall be removed. I am inclined to think it is so large now that it would be the better plan to sacrifice the eye. The operation should be done with great promptitude. I advised it some three weeks ago, but the patient is still undecided. If it is done now we may still, perhaps, be able to do thorough work, which will surely be impossible shortly.

In conclusion, I shall be pleased to have any of the gentlemen present examine the patient if they desire.

NOTE.—As a matter of interest let us add that the following day after this meeting Professor Merple examined the eye by my request and urged immediate removal of it (together, of course, with the tumor in the outer side of the orbit), fearing that otherwise a sympathetic involvement of the other eye would appear and result in total blindness. This operation I accordingly performed without delay. At the present time (some two months later) there has been no return of the growth reported, and the patient has gained both in weight and color. But the prognosis as to the final recurrence is very bad, when the roots of the malignant disease have had time, before operation, to become so wide-spread as in this patient was the case. During her presence before you I could hardly allude to this fact; but it is the main lesson to be learned, and the chief reason for presenting this lady to you. No reproach clings to her physician, Dr. Freudenthal, whose action was a prompt one in urging surgical intervention. But months earlier her dentist had opportunity to observe suspicious signs of antrum involvement, and maxillary disease. I am sure that no member of this Institute would, under like circumstances, neglect, upon the mere suspicion, to save a human life by at least calling for an immediate surgical consultation.

*Dr. C. O. Kimball.*—I wish to present to the society the apologies of two of our members: first that of Dr. S. A. Hopkins, of Boston, who is very much interested in the paper of the evening, and who had planned to be here expecting to take part in the discussion; but he says he cannot leave because his little step-son has typhoid fever. The other is from our friend Dr. Andrews:

### REMARKS ON THE PAPER OF M. MICHAELS.

BY R. R. ANDREWS, CAMBRIDGE, MASS.

The etiology of the chemical erosion of the teeth has been a most difficult, obscure, and unsatisfactory subject for the dental pathologist to investigate. And notwithstanding the considerable attention that has been given to it by able minds, we are as yet not wholly certain as to its actual cause. It would seem that from all the accumulation of data given us something tangible should have resulted. The paper of the evening, entitled "On the Rôle of Systemic Hyperacidity and of the Sulphocyanides in the Saliva in Chemical Abrasion of the Teeth," as it has been presented, although differing in theory from those of some of the more eminent investigators, is to my mind the most satisfactory and convincing of all. I confess that I would have preferred that the author use the word erosion rather than abrasion in his title, as the definition seems more significant.

So eminent authorities as Tomes and Salter, with very many others, regard erosion as the result of too vigorous use of a stiff tooth-brush, and as being caused alone by friction. But it is not an abrasion of this kind. Magitot regarded the trouble as true caries, and stated that these defects of the teeth were instances of healed caries of the neck of the tooth, or dry caries, without, however, adducing any reasons in support of this theory. Fox speaks of this defect as the removal of the enamel which was not produced by caries. He says it occurs upon the labial surfaces of incisors particularly, which appear as if they had been gnawed. And Fox's description is in accord with the appearance of several different cases which I have had under my own observation within the last few years. These cases are not the wedge-shaped depressions so commonly met with, where the edges are so well defined, but the whole labial surface of the enamel of the superior incisors looks, as Fox has stated, as though it had been gnawed, the deeper depressions being nearer the gums, the whole surface being beautifully polished, but without any definite edges. The only instance which I have ever heard of or seen, of what I suppose to be this same pathological condition of the lingual or tongue surfaces of the teeth, was shown to me by my friend, Dr. L. D. Shepard, of Boston. The patient had lost almost the entire layer of enamel from the lingual surfaces of her inferior cuspids and incisors. They were very much eroded and beautifully polished. The case was so marked that there seems very little doubt but that it was a true case of erosion on this portion of the teeth. Some have advanced the theory that erosion is caused by acid currents of saliva in the mouth passing over the teeth near the gum margin, and that it is this constant current which is responsible for the wearing away of the enamel at this point.

Dr. Black was able to form grooves in teeth artificially by the corroding action of a mild solution of sulphuric acid in water, the outer whirl of the revolving current of this solution causing in time a deep groove in the enamel and dentine of a bicuspid tooth.

Under the microscope the appearance of the section of an eroded tooth is in every way unlike the appearance of true caries. It has the same general appearance as sections of mechanically abraded teeth. Associated with this we may, or may not, find secondary dentine forming in the pulp-chamber. At times this secondary growth is very considerable, sometimes entirely absent. Towards the eroded part of the section we find a peculiar appearance, next that portion of the dentine which is exposed by the erosion. This has been called a *zone of resistance.* It has been the subject of much investigation, and its appearance is undoubtedly caused by myriads of minute, globular, glistening bodies filling the ends of the dentinal canals nearest the eroded surface. Dr. Black considers these globules to be fat globules, produced in the canal by the decayed dentine fibre, but in these cases there are no decayed dentine fibres,—The dentine and its fibres are alive. I have always held Dr. Black to be in error in regard to these globules. What really takes place in the dentinal canals where the surface is eroded and irritated may be thus described: An irritation to an exposed dental surface causes the pulp to forward to the injury, through the canals involved, large numbers of lime globules,—calcospherites. These are found near the injury. They can always be found by using the higher powers of the microscope. It is probable that the globules never calcify in the canals, but of this we are not certain. Robert Arthur claimed that they did, and that the new surface produced never, or seldom ever, afterwards decayed. That the surfaces under discussion are protected from infection is known to most of us. In vital dentine nature always makes this effort to protect an injury. Secondary dentine is formed for the same purpose at the pulp end of the canals.

If this secondary formation of dentine is excessive, it may, and probably will in time, end in the death of the pulp.

A point well worth remarking in the paper of Dr. Michaels is the statement, "No matter how feeble the dissolved chemical element may be, if it have affinity for a base, there is a reaction."

In this statement he must mean "dissolved chemical element" to be of, or have, acid properties, in order to have affinity for a base. With this interpretation, we have a simple chemical law expounded, which is too little appreciated.

The statement is made in my translation that "the alkaline sulphocyanide (of potassium and ammonium) dissolves the *ossien* of the teeth, exposes their mineral elements, and unites with them to form sulphocyanide of calcium, and soluble phosphates of potassium and and ammonium."

On looking up the word "ossien," I cannot find it. I find *ossium,* signifying "of the bones," and also find the word *ostein,* signifying "animal matter of bone," and I am in a quandary. Is it the fault of the translator?

If this alkaline double sulphocyanide salt of potassium and ammonium can attack and break down the calcic phosphate of the inorganic enamel, it is a fact hitherto, to my knowledge, unknown and very significant. The paper must be considered one of the most valued contributions to the subject of erosion that has ever been offered to our profession. The author's evidence seems conclusive, logical, and convincing.

I regret that I have not the chemical knowledge requisite to give the more critical attention which so able a paper deserves. I can only appreciate and applaud.

*Dr. J. Morgan Howe.*—This obscure subject is one which has interested me for many years. I remember several occasions on which it had been under discussion, when the question of a uric acid diathesis has been brought up, and the assertion made that chemical erosion is associated with this condition of the system, and again denied by others.

My interest in the subject led me several years ago to have a number of urinary analyses made from patients who had distinctly eroded places on their teeth, but who had little or no other manifestations of uric acid diathesis. One or two had occasional rheumatic twinges. One of the patients, a lady, denied that she had ever had any suggestion in her physical condition of any such trouble. The analyses were made by a well-known expert of New York, and in each case he reported that there was, without question, a large excess of uric acid. Pressure of business and other things prevented me from carrying the investigations farther, but so far they certainly bear out the statements made by M. Michaels, —that erosion is associated with, if not caused by, this uric acid diathesis. The local manifestations of erosion have appeared to me so various that I am unable to account for many of them by regarding them as the result of the action of the fluids exuded from glands in the mucous membrane directly upon the surface affected.

The determination of extent and location of the wasting process by friction does not eliminate the chemical factor, but seems to show that the solvent quality is distributed throughout the oral fluids; although, on the other hand, I have seen linear grooves at the necks of the molar teeth from buccal to palatal side, as though cut with a rat-tail file, where friction could have had almost no effect.

We are much indebted to Dr. Michaels for giving us the results of his investigations. They point, as others have done, to a vitiated or abnormal condition of the glandular secretions, which we can only hope to cope with, from the stand-point of medical men, by seeking to produce such an effect upon the system as will cause these glands to resume normal action.

*Dr. Potter.*—There are a few words which I would like to say in addition. It is rather unsatisfactory to me that the author does not explain the connection between the sulphocyanides and the urates; why is it that when the urates are present the sulphocyanides are present in increased amounts?

Again, he does not explain the action of the urates in causing the erosion. I cannot find how the urates which at the time appear in the saliva accomplish the erosion. In a conversation with Dr. Hills, the assistant professor of chemistry of the Harvard Medical School, I propounded this theory to him, and asked him if he believed it chemically possible. He stated that he did not think there was enough of the sulphocyanides to produce an erosion. When I stated that it was not accomplished quickly, but might be extended over a period of five or six years, he said that it might be possible. I asked him if there was anything in the nature of a mouth-wash which would react against the sulphocyanides and thus remedy the evil? He said that he did not know of anything unless it was ferric chloride. I told him that that was worse than the sulphocyanides.

The subject of physiological chemistry, it seems to me, is worth dwelling upon right here. In the next term of the Harvard Dental School we are to teach the men physiological chemistry in order to enable them to deal with these conditions, and I do not think that any one who has not had a training in this subject can take hold of these problems with satisfaction. I find myself going back to text-books on urinary analysis as soon as I take hold of the subject. We have about reached our limit in mechanical operations. What we now want to perfect is the treatment of pathological conditions of the sockets of the teeth. We cannot be satisfied until we find a proper solution to questions of this sort, and they are to be found in a study of physiological chemistry. I want to mention a book which is of value if one is to make a study of the uric acid diathesis. It is called "Uric Acid in the Causation of Disease," by Haig. Dr. Haig is an extremist, and attributes many diseases to the heaping up of the urates in the tissues. I

do not think medical men generally follow him to the extremes to which he goes, but by reading his book many points are brought out which will serve to impress upon one the value of physiological chemistry.

*Dr. Kimball.*—I wish to express my personal thanks to Dr. Potter for the clear *résumé* of Dr. Michaels's paper which he has made. It has seemed to me that the theory which Dr. Michaels advances, so far as I understand it, better meets the conditions which we see than any theory of which I am aware. In these erosions of the teeth it seems to me that we should carefully distinguish those which are mechanical from those which show, from their position and shape, that they have apparently some other cause.

We are all familiar with the mechanical abrasion of teeth up at the neck, and we have all seen teeth which have been very much cut by the excessive use of tooth-powder. I remember a few years ago a gentleman came to me with a very disagreeable set of teeth, and I scolded him well about cleansing his teeth thoroughly without giving him any special directions. When I saw him a year later he had cut deep grooves above the enamel of bicuspids and canines on both sides of the mouth. I found he had been using a tooth-powder two or three times a day with a stiff brush. I stopped that, and the teeth did not cut away any more. This simply illustrates the rapidity with which these abrasions may occur.

In those forms of erosions of which he specially speaks, which from their situations preclude any rational explanation as excessive use of the tooth-brush,—the round, pit-shaped erosions which are frequently found on the front of the incisors,—there must be something either in the surface of the tooth which will make the enamel softer, or else something which exerts a solvent influence upon the surface in this spot. It seems to me that Dr. Michaels's theory of the secretions of the labial glands in this connection is very satisfactory. He has shown chemically that not only does this acid saliva contain the sulphocyanides of ammonium and potassium in comparatively large amounts, but he has found that similar solutions used outside the mouth upon teeth will produce similar loss of surface.

In regard to what Dr. Howe said about the erosions attacking the inside or palatine surfaces of the teeth, I would like to mention an instructive case which I observed a few years ago. It was the case of a lady whose teeth had been under my care for a number of years. Suddenly, at one of her annual examinations, I found that for some reason all the approximal fillings between the incisors were beginning to stand out above the surfaces of the teeth. This struck me as being rather curious, and upon investigation I found that the fillings were not loosened, but that the surfaces of the teeth were disappearing. The front teeth were losing their enamel on the inner surface as far back as and including the bicuspids, first and second, upper and lower. The enamel was nearly gone. The outer surfaces of the teeth were not affected at all. I found that she was taking hydrobromic acid for a headache, without any precaution. I obtained a sample of the same strength as that which she was taking; into this I placed an incisor tooth which had previously been partially covered with wax, and in twenty-four hours all the enamel which had not been so protected was eaten off.

*Dr. H. S. McNaughton.*—I wish to present several casts of teeth in an eroded condition. The first cast is that of a central and a lateral incisor, showing a groove on the labial surface of the central. The lateral was discolored, showing disintegration, which may be a preparatory stage of the erosion.

This second cast is from the mouth of a young woman of twenty-five, who had retained the two temporary superior cuspids. One of these teeth had had the crown entirely cut off by erosion, the other one nearly so. The impression was taken before the extraction of the teeth, and then the teeth were placed in the impression, so that the cast shows the teeth as they were in the mouth.

Another cast shows a lateral incisor in which there was a large labial cavity. When the case first presented, the tooth had a bright pink appearance, as though there might be a drop of blood under the enamel. On examination I found that there was a large cavity filled with an excresence of gum; this was removed, and the walls of the cavity were found to be as hard as if cut from a sound tooth. The cavity was filled with gutta-percha. This did did not affect the dissolving process, the tooth breaking off at this point after two years.

Another cast shows erosion on the lingual surfaces of all the molars and bicuspids. Casts of upper and lower teeth of the same case, occlusion nearly normal, show erosion on all sides of the anterior teeth.

I wish also to call attention to the fact that the thick labial mucus may assist in causing erosion without itself being the solvent, by spreading over the surfaces of the teeth, forming an almost insoluble coating, which holds any destructive secretions from the margin of the gums and keeps out the alkaline saliva.

*Dr. T. P. Hyatt.*—I should like to mention a case which came under my observation a very short time ago. The patient was a lady whose mouth I had thoroughly examined, and the teeth had all been put in good order. There were no signs of erosion previous to her starting South to spend several months. After she had been away about four months I received a letter from her stating that there were two cavities on the labial surfaces of her two superior central incisors. She said that she had called on three dentists, and that they had all advised gold fillings. I wrote to her not to have the gold fillings on account of the disfigurement, and asked her if possible to come to New York and let me examine the teeth, as I could not understand cavities from decay occurring in so short a time. I also, in my letter, advised her to use Phillips's milk of magnesia. She came on, and to my astonishment I found two well-defined abrasions upon the superior central tooth, about one-eighth of an inch from the margin of the gum, oval in shape, and three-sixteenths of an inch broad at the widest part.

I treated them by drying the surfaces and then applying carbolic acid crystals evaporated with hot air, and advised the patient to use milk of magnesia. Since that time the teeth have become no worse. It would be a stretch of imagination to say that they were any better, but I will say that the edges have become less sharply defined.

I should like to ask the question, Is it possible that there are medicines which would cause these abrasions? If so, why only on one or two teeth; or is it likely that they were due to some constitutional derangement?

*Dr. Louis C. Leroy.*—It is a deplorable fact that there are so few chemical experts in the dental profession who are able to render intelligent scientific opinions or discuss subjects of a chemical nature. As Dr. Potter has said, we need a special education in that department to be able to study a subject of this kind. Erosions of the teeth, such as have been presented to us to-night and which we may find in many mouths, I believe to be not of mechanical but of chemical origin, and distinct from mechanical abrasions. They evidently start from a chemical source, although possibly assisted by mechanical means. Fillings may stop the process for a time, but the erosion will very probably continue around the margin, and in a short time the filling will have to be repaired. I have endeavored to account for this in various ways. I have observed it more commonly in persons of excessive nervous temperament, weak mentality, and cerebral troubles. This point is mentioned as of possible interest at the present moment, although undoubtedly all have noted the same circumstance.

*Dr. F. Milton Smith.*—The topic before us is one which interests me very much. I never feel my helplessness quite so much as when I have a bad case of pyorrhœa or a case of this kind. I have to be honest sometimes and tell my patients that I do not know what to do for them. I remember having heard what was to me an exceedingly interesting paper upon this subject, read before the New Jersey State Dental Society, about six years ago, by Dr. Winkler, of New York. While he did not go so deeply into the subject as does Dr. Michaels, as I remember, the ground he took was that erosions of the teeth are caused by a secretion from these labial glands. He claimed that he got very excellent results by administering minute doses of creosote. I should like to inquire if any of the gentlemen present heard the paper, or have made any experiments along this line.

*Dr. Leroy.*—I think the paper to which Dr. Smith refers was on the subject of caries (Addendum, New Jersey State Dental Society Proceedings, 1895, p. 54),—"The Medicinal Prevention of Dental Caries," by Dr. George Howe Winkler, D.D.S., of New York.

*The President.*—I was pleased to hear Dr. Leroy so carefully discriminate between the words "abrasion" and "erosion." I take occasion just here to state that Dr. Michaels uses these two words indiscriminately. So far as I understand Dr. Michaels, he has reached the conclusion that there is a balance in the system which if preserved means health, and if not preserved means disease more or less marked; that the retrograde metamorphoses which are constantly going on in disease are frequently brought about by some simple failure on the part of the system to properly respond to the breaking-down and building-up processes. Following that line of reasoning, that any deviation from a complete balance is disease, he was not long in analyzing the saliva and testing each one of the elements contained in normal saliva to see if either one could be responsible for erosion. Several years ago—I should think four or five—I saw his first experiments, which he alludes to here very casually. It was a very nice experiment, and consisted of two bottles, one containing a little distilled water into which had been put one milligramme of sulphocyanide of potash. This was furnished with a glass siphon drawn to a point at the lower end, so that the solution would fall a drop at a time onto a tooth; the other siphon was made small at its short end, and was arranged to touch the tooth and carry the solution away, so that nothing was spilled. In a little time a tooth subjected to this process was eroded. I repeated this experiment here in New York, and I think some of the gentlemen present saw my rough imitation of Dr. Michaels's apparatus and saw the slight erosions produced by this solution of sulphocyanide.

Starting upon this rather slender basis, as he acknowledges himself, Dr. Michaels next caused analyses to be made of the blood, saliva, urine, and sweat of all the patients he could lay his hands on who had distinctly marked erosions. In all of these cases he observed an increase of sulphocyanide, and in one it amounted to five hundred per cent. He told me about this case, and said that the patient, like all the others, was a sick man. I told him of a case that I had had under my observation for ten years in New York, and that the erosion was growing. He replied that he should use the cautery and burn out these ducts, at least, if not the glands. I have not had the courage to do that. I have noted in these cases a marked derangement in the general health, but I have not gone to the extent of making an analysis of the saliva or the excretory fluids.

It seems to me that Dr. Michaels leaves us rather confused in speaking at one time of the saliva containing the large increase of sulphocyanide, and again speaking of the secretion from the labial glands as containing the excess of sulphocyanide and being responsible for the erosion. I think this point might be rectified, and it should be made more clear whether the contents of the saliva or the products of the glands does the mischief. Be that as it may, he has certainly opened up a field for our investigation which should not be closed again right away. I also think we all owe our thanks, individually and collectively, to Dr. Potter for the very careful and accurate epitome on the subject. I shall be very glad if he will close the discussion in summing up what has been said, and making such reply as he desires.

*Dr. Potter.*—I think I have said all I wish upon this subject.

It is one of those subjects upon which none of us can speak authoritatively. I feel as if the writer has entered upon a very interesting and profitable field of observation, and that it is important that we follow it up to a satisfactory conclusion. While I do not think the paper accounts for all the causes of erosion, still it gives us an incentive to further investigation.

Upon motion, a vote of thanks was extended to Dr. Potter for his kindness in presenting the subject to the Institute, as well as to Dr. Dawbarn for presenting the case of sarcoma of the antrum. The Secretary was also instructed to convey to Dr. Dawbarn's patient the condolences and thanks of the Society for her affliction and her kindness in consenting to appear before the meeting.

Adjourned.

FRED. L. BOGUE, M.D., D.D.S.,
*Editor The New York Institute of Stomatology.*

